# Fluorescent Fluid in 3D‐Printed Microreactors for the Acceleration of Photocatalytic Reactions

**DOI:** 10.1002/advs.201900583

**Published:** 2019-04-26

**Authors:** Lijing Zhang, Zhigang Zhu, Bofan Liu, Chong Li, Yongxian Yu, Shengyang Tao, Tingju Li

**Affiliations:** ^1^ Department of Chemistry Dalian University of Technology Dalian 116024 P. R. China; ^2^ School of Materials Science and Engineering Dalian University of Technology Dalian 116024 P. R. China

**Keywords:** 3D printing, fluorescent fluids, light‐converting media, microreactors, photochemistry

## Abstract

The photochemical microreactor has been a burgeoning field with important application in promoting photocatalytic reactions. The integration of light‐converting media and microflow chemistry renders new opportunity for efficient utilization of light and high conversion rate. However, the flexibility of emission light wavelength regulation and the universality of the microreactor remain significant problems to be solved. Here, a photochemical microreactor filled with fluorescent fluid is fabricated by a 3D printing technique. The light‐converting medium in the fluorescent fluid is used to collect and convert light, and then delivers light energy to the embedded continuous‐flow reaction channels to promote the chemical reaction process. With the merits of flowability, different light‐converting media can be replaced, making it a general tool for photocatalytic reactions in rapid screening, parameters optimization, and kinetic mechanism research.

Light‐harvesting and utilization are essential ways for the growth and reproduction of plants and animals. Most biochemical reactions in an organism are carried out under the introduction of light, such as plant photosynthesis, vitamin conversion in the human body, skin melanin deposition, bacteria disinfection, and so on. In the process of using light, the organism has evolved a variety of sophisticated and efficient methods for energy transfer in microscale, which can strengthen the absorption and conversion of light energy. Inspired by nature, human beings have developed many kinds of photoreactors to promote various photochemical and photophysical processes. For example, the photonic crystal was used to enhance the light utilization in solar cells and light‐emitting diode (LED) devices,^1^, ^2^ Fresnel lens was used for light concentration,^3^, ^4^, ^5^ the microchannel is used to strengthen the effect between fluid and photocatalyst,^6^, ^7^ and the optofluidic was used to control the light transmission.^8^, ^9^, ^10^ Recently, the combination of a continuous‐flow microreactor and the photochemical reaction has attracted widespread interests among chemists. Continuous‐flow microreactor renders high surface‐to‐volume ratio (i.e., small channel depth) and enhanced heat and mass transfer efficiency as compared to conventional batch systems, which can significantly improve the yield and selectivity of synthetic products.^11^, ^12^ Moreover, microreactor enables shorter light path and more uniform light irradiation as compared to traditional batch reactors when it is located under an external light source, and can effectively overcome the limitation of Lambert–Beer law.^13^

However, in the photochemical reaction, due to the intrinsic limitation of the basic principle of quantum mechanics, its occurrence and conversion are directly determined by the wavelength of the external radiation source. Thus, light source with a specific wavelength is a critical factor for photochemical reactions.^14^ In reality, some light sources with particular wavelength are costly or hard to obtain, which severely restricts the progress of many photochemical reactions. As is known, during photosynthesis, the chromophore system in leaves can harvest sunlight and convert it to the specific energy needed for chemical species synthesis.^15^, ^16^ Inspired by the chromophore system, light‐harvesting and converting media provide a good idea for solving the problem of the specific wavelength. Many luminophores (e.g., fluorescent dyes or quantum dots) have the characteristics of broad wavelength absorption and narrow wavelength emission.^17^ By using this feature, people can utilize low‐cost LED or even sunlight to initiate photochemical reactions. A requirement for the light‐converting media (i.e., luminophore) is that its emission profile should match with the maximum absorption of the photocatalyst or photosensitizer used in target photochemical reaction. Debije and Noel introduced Lumogen F Red 305 (LR305) dye in polydimethylsiloxane to simulate the light transporting properties of the leaves and developed a highly efficient continuous‐flow luminescent solar concentrator photomicroreactor (LSC‐PM) which could work under natural sunlight condition.^18^, ^19^, ^20^ Since the dye of LR305 has been doped into the reactor matrix, it cannot be changed, so this type of reactor is only suitable for the photochemical reactions initiated by LR305 emitting‐light. At the same time, the doped dyes cannot be separated and recycled. All these will lead to an increase in the manufacturing cost and the decline in the applicability of the LSC‐PM. Therefore, the development of cost‐effective and general‐used light‐converting photomicroreactor^21^ is of great significance to the wide and standardized application of photomicroreactors in the field of photochemistry.

Such microreactors should have the following characteristics: 1) the distribution of light‐converting media in the 3D space is controllable to produce a homogenous light field; 2) the light‐converting media in the reactor should be replaced flexibly to match various photochemical reactions in the reaction channel, so as to broaden the application range of the reactor.

In order to fulfill the requirements mentioned above, we pioneered a general‐used fluorescent fluid photochemical microreactor (FFPM). Such an FFPM includes two parts. One is the light channel, which is filled with fluorescent fluid, and the other is the reaction channel, where the reaction substrate and catalyst solution are injected through the syringe pumps. Light‐converting medium in the fluorescent fluid can capture ambient light of different wavelengths and convert them into emission light with narrow wavelength. Then the emission light will be transferred to the embedded reaction channel, thereby affecting the chemical reaction process. A schematic illustration of the FFPM system and light transfer process is shown in **Figure** **1**a.

**Figure 1:**
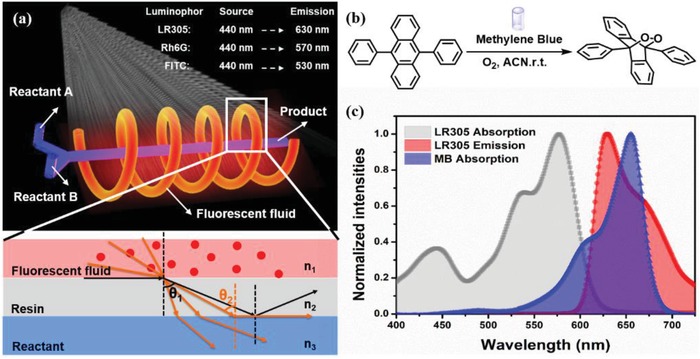
a) Schematic illustration of the microchannel structure in FFPM and the light conversion process from the fluorescence fluid to reaction liquid. b) The cycloaddition of DPA to the endoperoxide was used as a model reaction. c) Spectra overlapping scheme of the LR305/MB which provides enhanced photon flux needed for the reaction channel.

In such an FFPM system, the light‐converting medium was introduced into the light channel as fluid, which presents many unique merits compared with the solid dopant. First, most of the luminophores have better solubility and dispersibility in organic solvents, and exhibit equivalent or higher fluorescence transmission efficiency than that in the traditional solid matrix under diffuse light.^22^ Moreover, fluids can adjust the property of emission light by merely replacing a different luminous body. Also, due to flowability, fluids can be easily shaped. Through the structural design of the light channel, the distribution of fluorescence fluid in 3D space can be controlled, thus affecting the spatial distribution of the light field. Compared with LSC‐PM system, the FFPM is more flexible and controllable, which will further expand its application in the field of photochemical reactions.

The structure design and spatial distribution of the light channel and reaction channel are the key factors affecting the final performance of the FFPM. Due to the complexity of channel structure, the conventional methods, such as soft‐lithography^23^ and print‐and‐peel techniques^24^ are difficult to realize. 3D printing is an emerging additive manufacturing method, which can build objects with specified structure through layer‐by‐layer depositing materials.^25^ With the aid of computer‐aided design, 3D printing can conveniently fabricate the FFPMs with light channels and reaction channels of different structures. After the microreactor was fabricated, fluids containing fluorescent dyes were injected into the light channel to perform the functions of light harvest and wavelength conversion, resulted in a complete FFPM (see the Supporting Information).

The cycloaddition of 9,10‐diphenylanthracene (DPA) to the corresponding endoperoxide (Figure 1b) was selected as a model reaction since it displays light‐limited kinetics and almost entirely independent of temperature.^26^ In this reaction, methylene blue (MB) was used as photocatalyst with a maximum absorption peak at 654 nm, and LR305^27^ was chosen as a light‐converting medium. The perfect overlap between LR305 emission and MB absorption (Figure 1c) was expected to provide enhanced photon flux needed for the reaction channel. The analysis of reaction kinetic was also performed to evaluate the acceleration of this reaction. Furthermore, the reaction rate constants at different luminophore concentration were calculated, which has specific guiding significance in reactor structure design and the concentration selection of luminophore.

First, we optimized the structure and shape parameters of the light channel to obtain the optimal light field. Three light channels were designed, which are a helix, linear array, and cylinder. While the reaction channel was fixed as Y type with a cross‐section of 1 × 1 mm^2^. The illuminance distribution of the light field received by the reaction channel was analyzed based on the Monte Carlo ray‐tracing method.^28^, ^29^, ^30^ The distribution of light channels and distance between light channel and reaction channel were also optimized to enable more fluorescent photons transferred to the reaction channel (see the Supporting Information). Notably, the shape and distribution of the light channel in FFPM remarkably influence the illuminance received by reaction channel (**Figure** **2**a). The cylindrical channel shows the highest illuminance intensity, which may be due to the larger volume of the fluorescent fluid and more uniform distribution of fluorescent fluid around the reaction channel. We also considered the case of full coverage of the linear array. Although the illumination is similar to that of the cylinder, its uniformity is not as good as that of the cylinder (Figure S6, Supporting Information). The corresponding experiment results in Figure 2c also show that the cylindrical channel achieves the highest conversion of DPA (about 27%), indicating this kind of light channel induced more photon flux to the reaction process. Besides, compared with the results of 0 ppm (<5%), the promotion in the DPA conversion rate also proves that the introduction of fluorescence fluid light field can indeed accelerate the reaction process.

**Figure 2:**
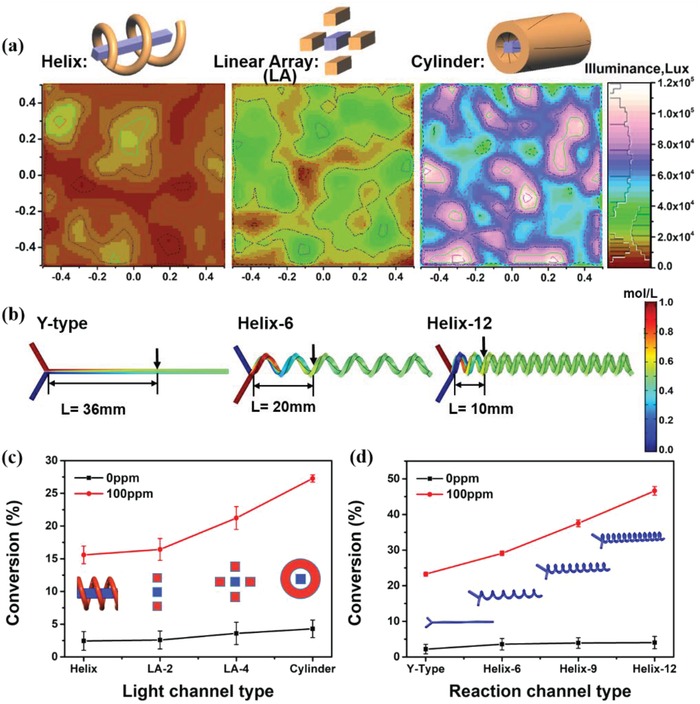
a) Illuminance distribution on the central cross‐section (1 mm^2^) of the reaction channel illuminated by different shape fluorescent fluid sources. b) Concentration field of numerical simulation analysis of different reaction channels, the optical channel is cylindrical. c) The effect of the light channel structure on the reaction conversion rate, the reaction channel is fixed as a Y type. d) The effect of the reaction channel structure on the reaction conversion rate (condition: blue LED with an emitting peak at 440 nm, 20 V; flow rate at 50 uL min^−1^).

Next, we conducted reaction channels screening using the finite element method (Figure 2b).^31^, ^32^ Compared with a straight channel, the helical reaction channel is better for diffusion and mixing. For the helical channel with 12 turns (Helix‐12), the mixing path is shortened for nearly 3.6‐fold, which helps strengthen the interaction between reactants and increase the reaction time. Also, the helical reaction channels have greater flexibility in spatial configuration and longer route (i.e., residence time) when the overall length of microreactors is fixed. The experimental result in Figure 2d also confirmed our guess. Thus, the photomicroreactor with the cylindrical light channel and helical reaction channel was selected for further study. The specific structural parameters of each FFPM are listed in Tables S1 and S2 in the Supporting Information.

To demonstrate the convenience and flexibility of the FFPM used in DPA oxidation, different reaction conditions were optimized, such as the luminophore concentration, light input power, and residence time (i.e., flow rate). The most outstanding feature of FFPM is that it can convert light of any wavelength into the wavelength required for the reaction. Here to highlight the wavelength conversion function of fluorescent fluids, we randomly selected a cheap blue LED with an emission peak of 440 nm, which mismatch to MB's absorption spectrum. The price parameters for different LED were listed in Table S3 in the Supporting Information. First, the FFPM with different LR305 concentration can be easily obtained by only changing the dye concentration from low to high, and their performance on the conversion of DPA under different applied voltage was recorded (**Figure** **3**a). The reactor without LR305 showed low conversion no more than 20% even when the LED strip worked under full voltage. While reactors with LR305 showed a corresponding increase in conversion as the voltage increased, clearly indicating the effectiveness of wavelength conversion induced by LR305. The FFPM with 400 ppm LR305 shows the highest conversion of 70% at 23 V, resulting in a 3.5‐fold increase compared with 0 ppm. Figure 3b shows the effect of the residence time on the conversion at 20 V. In all reactors, prolonging the residence time is conducive to the conversion of DPA. Under the same retention time (86 s), the introduction of fluorescent fluids resulted in a better conversion rate. With the LR305 concentration increased from 0 to 400 ppm, the conversion rate increased for nearly four times. These results show that this kind of FFPM renders energy and time efficient way to promote the transformation of reactants, which is mainly due to the increased intensity of photon flux caused by wavelength conversion (blue light to red light).

**Figure 3:**
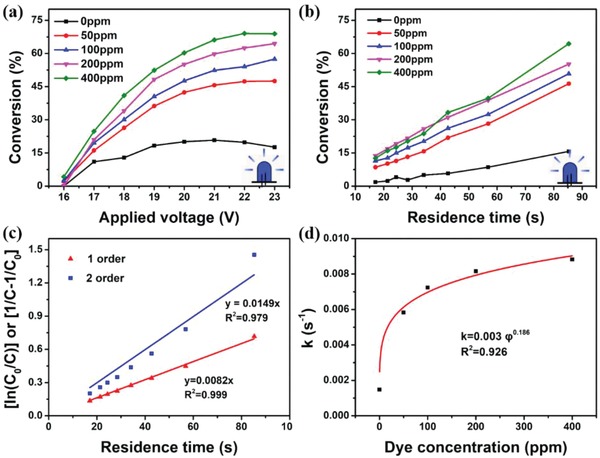
Reaction conditions screening and reaction kinetic research. a) Effect of LR305 concentration and applied voltage on the DPA conversion under a flow rate at 50 uL min^−1^. b) The relationship between the DPA conversion and the residence time at different LR305 concentration under 20 V blue LED irradiation. c) Validation of the reaction order with respect to the DPA (20 V blue LED, 200 ppm LR305). d) The relationship between the reaction rate constant *k* and LR305 concentration.

The increased photon flux caused by the fluorescent fluid can accelerate the apparent reaction kinetics. Reaction order concerning DPA was further investigated. In this case, the oxygen concentration in the reaction mixture maintained saturated. Figure 3c shows the relationship between DPA concentration and reaction time. Based on the analysis, it can be concluded that the photocatalytic oxidation of DPA is first order concerning DPA. The rate equation is ln(*C*_0_/*C*) = *kt*, where *k* is the apparent reaction rate constant about DPA. In this reaction system, *k* includes information on the intrinsic reaction rate constant and the concentration of the fluorescent fluid. Its value increases with the increase of LR305 concentration. Figure 3d gives a more accurate nonlinear relationship, *k* = 0.003*f ^^^0.186, where *f* is the concentration of LR305. A similar relationship of *k* = 0.002*f ^^^0.289 in the serpentine reactor has also been derived (Figure S14 in the Supporting Information). Thus, an empirical formula of *k* = a*f ^^^ *b* was inferred, where *a* and *b* can be defined as structural factors, which may be related to the spatial distribution of the light and reaction channels.

Furthermore, the performance of this FFPM worked under white LED was also investigated (Figure S15, Supporting Information). Under the irradiation of white light, the conversion rate of DPA in FFPM was still improved by 24%. It indicates that the LR305 in the fluorescence fluid can convert the non‐650 nm part of the white light into 650 nm to contribute the additional conversion rate. The performance of FFPMs under white LED provides a reference for its utilization of broad‐spectrum light source or even natural sunlight. Moreover, the performance of FFPM under simulated solar light source was investigated (Figure S16, Supporting Information). When illuminated by CEL‐HXF300 with 1.0 sun intensity, the FFPM with 400 ppm LR305 shows an increase of 35% in DPA conversion than FFPM with 0 ppm at a residence time of 15 s. The introduction of fluorescent fluid significantly improved the utilization of solar energy in reaction of DPA oxidation, which is significant for promoting photochemical reaction with sunlight as the energy source.

Also, the general applicability of the FFPM in different photochemical reactions is demonstrated. Due to the light channel and reaction channel are separate, both fluorescent fluids and reactants can be replaced as required. **Figure** **4**a demonstrates the dye replacement situation for DPA oxidation, which shows the possibility for the screening of light‐converting media. Both Rh6G and Eosin Y can also effectively harvest and convey photons to the reaction media, but the photon transmission efficiency is far less than that of LR305, while the introduction of fluorescein isothiocyanate (FITC) can hardly increase the conversion of DPA. It is determined by the overlapping degree of their emission to MB's absorption (Figure S17 in the Supporting Information). Figure 4b,c shows another photochemistry reaction of p‐thiocresol oxidation, which was conducted in this FFPM system with Eosin Y as photocatalyst and FITC as the luminophore, and under the illumination of blue LED.^[3b]^ 50% improvement in conversion was achieved with 400 ppm FITC.

**Figure 4:**
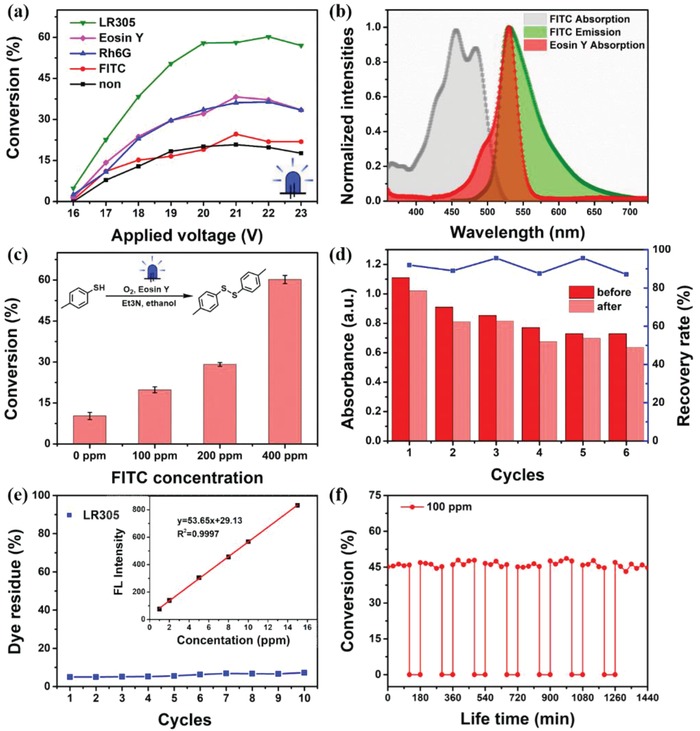
a) The effect of different dyes on DPA conversion; b) spectra overlapping scheme of FITC/Eosin Y; c) enhanced conversion for p‐thiocresol achieved by our fluorescent fluid microreactor; d) the absorbance change of LR305 before and after use and the corresponding recovery curve; e) dye residue on the inner surface of light channel, the inset shows the standard curve between fluorescence intensity and LR305 concentration with *R* ^2^ = 0.9997; f) lifetime study of this photochemical reactor.

The cost of printing such a photoreactor is less than $5. Most of the money is spent on light‐conversion media. Some fluorescent materials are extremely expensive (Table S4 in the Supporting Information), so the recycling of fluorescent materials is of great significance. Figure 4d shows the recovery and reuse situation of LR305. There is no significant decline in absorbance of LR305 before and after use, the recovery rate is more than 85% for six cycles, making our FFPM cost‐effective and eco‐friendly. At the same time, dye residues on the inner surface of the light channel were also studied, and less than 8% dye residue was observed after 10 cycles (Figure 4e). The low dye residue allows the reactor reuse under different dye fluids without obvious interference to each other.

Finally, the lifetime of the FFPM was studied. Taking the DPA reaction as an example, and per 2 h rest for 1 h as one cycle, the reactor could be operated continuously for nearly 1 day. The tolerance of photosensitive resin to different solvents was investigated (see Table S5 in the Supporting Information). For most commonly used solvents, such as water, toluene, isopropanol, ethanol, and acetonitrile, the photosensitive resin can work continuously for about 4 days. Thus, our FFPM can be applied to various photochemical reactions in different solvents.

In summary, we proposed a general‐used fluorescent fluidic photochemical microreactor by 3D printing technology. Due to the introduction of fluorescent fluid, enhanced photon flux was focused on the reaction channel through light‐harvest and wavelength‐conversion, and significant acceleration in conversion rate and apparent reaction kinetics was observed. Moreover, the flowability of the fluid enables flexible replacement of light‐converting media and reuse of the reactor and in doing so provides a low‐cost and eco‐friendly design. This strategy will find extensive application in photochemical mechanism study and valuable chemical compounds production for its operational convenience and flexibility, such as pharmaceuticals, pesticides, and fine chemicals.^33^

## Conflict of Interest

The authors declare no conflict of interest.

## Supporting information

Supplementary
