# Supplementary material for: Fluorescent Fluid in 3D‐Printed Microreactors for the Acceleration of Photocatalytic Reactions
Source: Adv Sci (Weinh). 2019 Apr 26;6(13):1900583. doi: 10.1002/advs.201900583 (PMC6662095; doi:10.1002/advs.201900583)
Supplement: Supplementary file 1 — Supplementary [file ADVS-6-1900583-s001.pdf]

Copyright WILEY-VCH Verlag GmbH & Co. KGaA, 69469 Weinheim, Germany, 2019.

ADVANCED SCIENCE
Open Access

Supporting Information

for *Adv. Sci.,* DOI: 10.1002/advs.201900583

Fluorescent Fluid in 3D-Printed Microreactors for the Acceleration of Photocatalytic Reactions

Lijing Zhang, Zhigang Zhu, Bofan Liu, Chong Li, Yongxian Yu, Shengyang Tao, and Tingju Li*

Copyright WILEY-VCH Verlag GmbH & Co. KGaA, 69469 Weinheim, Germany, 2019.

Supporting Information

Fluorescent Fluid in 3D-Printed Microreactors for the Acceleration of Photocatalytic Reactions

Lijing Zhang, Zhigang Zhu, Bofan Liu, Chong Li, Yongxian Yu, Shengyang Tao, Tingju Li*

Dr. L. Zhang, Z. Zhu, B. Liu, C. Li, Y. Yu, Prof. S. Tao

Department of Chemistry, Dalian University of Technology, Dalian, 116024, P.R.China

E-mail: taosy@dlut.edu.cn

Prof. T. Li

School of materials science and engineering, Dalian University of Technology, Dalian, 116024, China

Keywords: 3D printing, microreactor, light-converting media, fluorescent fluid, photochemistry

1. The production of 3D printed photochemical reactor

For the production of a 3D printed photomicroreactor, the computer-aided design (CAD) of the reactor was first designed by using Solid works software and converted to a G-code file via the PreForm 2.5.0 software. Then the structure was printed by a Formlabs Form 2 3D printer (Formlabs Tech Co., Ltd. America). After printing, the reactor model was removed from the working platform and immediately transferred into a wash tank for cleaning. The residual resin inside the channels should be extracted with a syringe and rinsed with copious ethanol to ensure both the light channel and reaction channel unobstructed. Subsequently, the reactor was placed under a UV lamp for photocurable treatment. Then fluorescent fluid of

different dyes with different concentrations was injected into the light channel as media for light harvest and wavelength conversion, thus resulted in the final photoreactor. The fluorescent fluid can be flexibly injected in or pumped out for replacement and recycling. The schematic overview of the fabrication process was shown in Figure S1. The commercial clear photosensitive resin (Formlabs Tech Co., Ltd. America) was selected as the printing raw materials by its transparency and moderate refractive index (1.51). The intrinsic absorption and fluorescence properties of this transparent resin were studied (Figure S2a) to ensure that no effect on photons transmission throughout the whole reaction system. The optimal curing time was also studied (Figure S2b). The transparency of the printed reactor decreases with the extension of curing time. Thus, shorter curing time should be selected while ensuring sufficient mechanical strength of the reactor. The final curing time of 4 h was selected. Some photomicroreactors with different channel structures were demonstrated in Figure S3. The specific structural parameters of the light channels and reaction channels were discussed in the main text are listed in Tables S1 and S2.

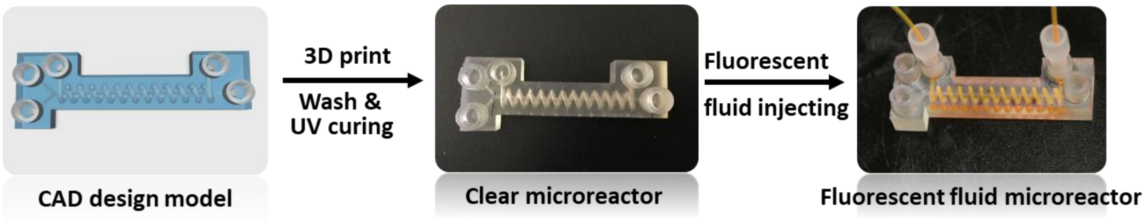

Figure S1. Fabrication process overview of fluorescent fluid photochemical microreactor (light channel: helical; reaction channel: Y type).

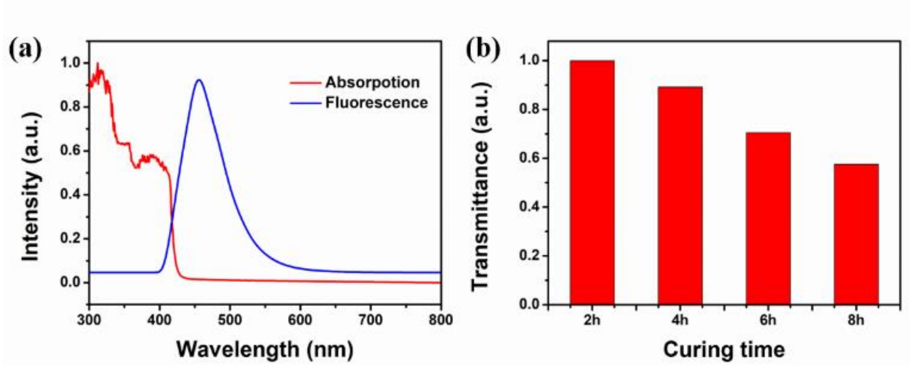

Figure S2. (a) The absorption and fluorescence spectra under 365 nm excitation of raw clear photosensitive resin; (b) The influence of curing time on the transparency of printed reactor.

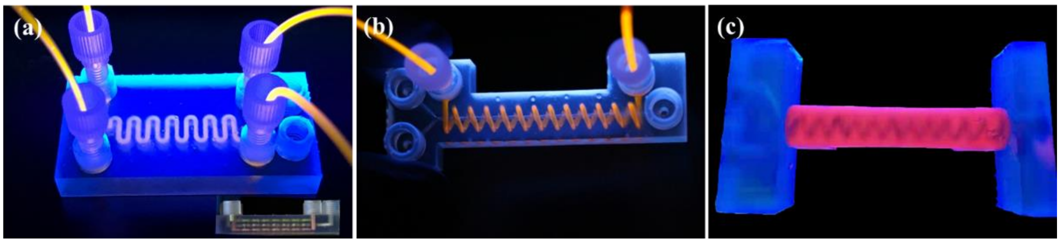

Figure S3. 3D printed photomicroreactors with different structures. (a) The serpentine reactor, both light, and reaction channels are serpentine; (b) Helical reactor, the light channel is helical, and the reaction channel is linear; (c) Cylindrical reactor, the light channel is cylindrical, and the reaction channel is helical.

2. Monte-Carlo Ray-tracing simulations

The Monte-Carlo Ray-tracing simulation was used to analyze the illumination situation of various types of FFPM we designed. In the simulation, the fluorescent fluids in the light channel are modeled as a volume source. To set up a model close to the real situation as much as possible, we set the linear-channel with $1\times1\times10$ mm^3 as one unit with luminous flux Φ = 1.00 lm. The flux of other light channels is set according to the relative volume. The central section of the reaction channel is set as the receiver plane ($1mm^2$). For each simulation, 1.5×10^5 rays are traced. Here are reported preliminary illuminance distribution results from the ray-tracing simulations.

The shape and structure selection for light channel

To evaluate the effect of the light channel shape and structure, three light channels of helix, linear-array and cylinder were designed, the specific structural parameters can be found in detail in Table S1. The cylindrical channel presents the highest average light flux illuminating of 0.0668 lm on the receiver plane and the illumination uniformity on this plane is 75.00%.

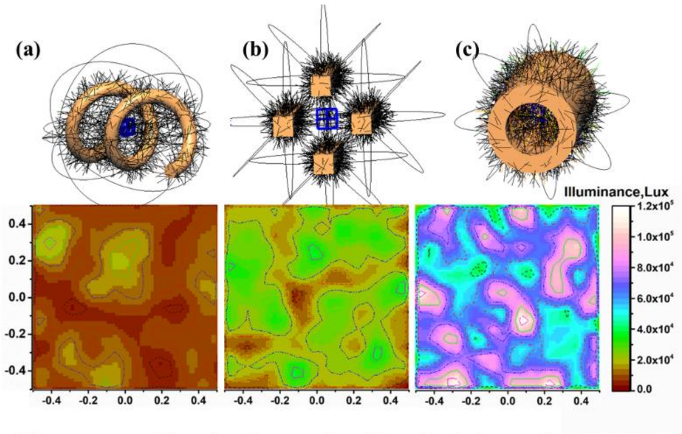

(d) Illumination and uniformity information

2487.3	11252	33493	Min.
22466	34258	115080	Max.
9879.4	21042	66866	Ave.
64.80%	71.80%	75.00%	uniform

Figure S4. Results of illuminance simulation for three different reactor designs with different light channel shape and structure.

Effect of the number of light channels and distance between light and reaction channel

The number and distribution of light channels around the reaction channel have a profound effect on the illuminance of the receiver plane. Moreover, the optical path of photons between the light channel and reaction channel will also affect the transmission of fluorescent photons. All of them are essential parameters. To gain insight into the effect of the number of channels within a certain FFPM, five reactors with increasing light channels number of 2, 4, 8, 12 and 16 were simulated, all light channels are symmetrically distributed.

Figure S5a shows the relationship between illuminance and light channel number, and Figure S5 c-g are the corresponding Illumination distribution images with contour lines. The illuminance increased with the increasing number of light channels, and the reactor with 16 channels (denoted as a square) display the highest light flux of 0.0669 lm which is similar to the cylinder one.

With the increased distance between the light channel and the reaction channel, the illuminance decreased dramatically. Figure S5b and Figure S5 h-l show the change tendency

and corresponding illuminance distribution. However, considering the precision of the 3D printer and the mechanical strength of the reactor, the final distance was selected as ∼1 mm.

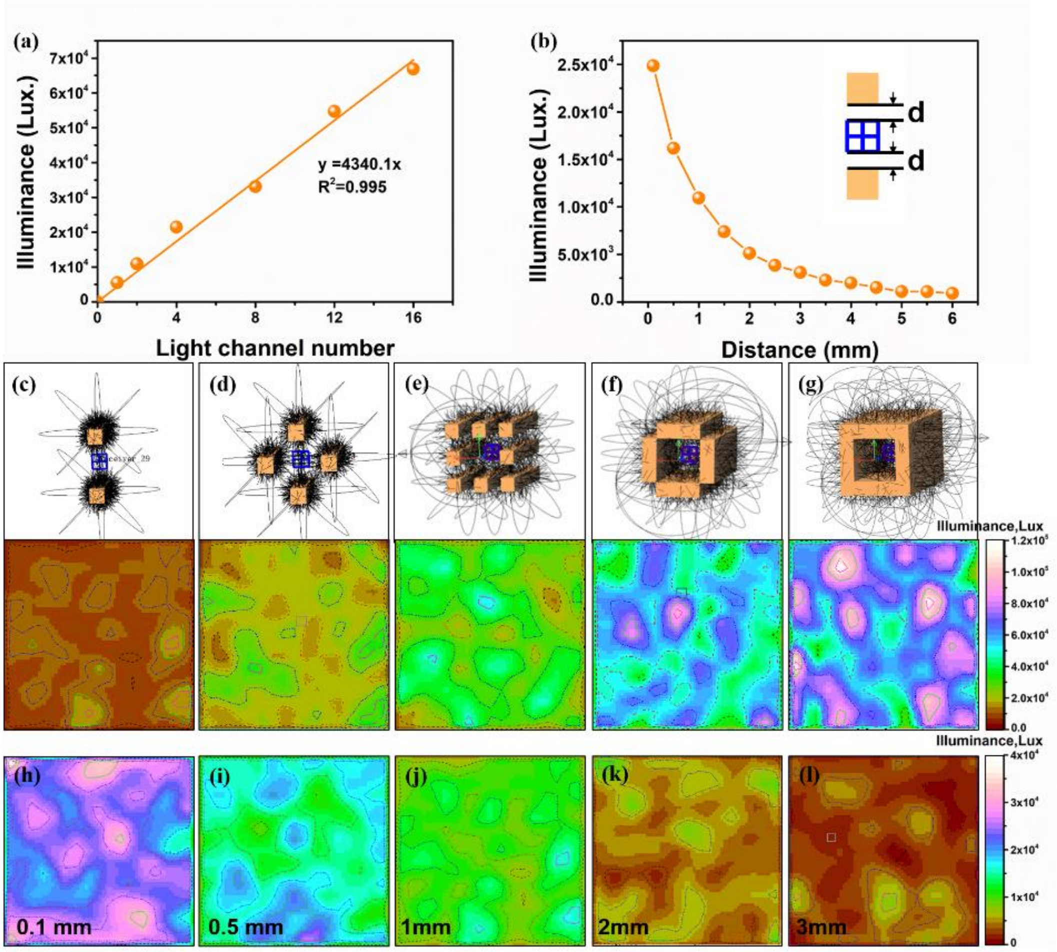

Figure S5. (a) Results of illuminance varying with the number of light channels; (b) Results of illuminance varying with the distance between the light channel and reaction channel; (c-g) Models of the designed FFPM with different number light channels and corresponding illuminance distribution with contour line. (h-l) Illuminance distribution images simulated from the two-linear channel with different distance.

Comparison of the linear array full coverage with the cylinder

Seen from Figure S4c and Figure S5g, the FFPMs with a linear array full coverage and cylinder channel have a similar intensity of illuminance, but the cylinder has much better uniformity. If we consider the distribution of one unit at different angles, we can see more intuitively that the cylinder has more uniform light flux. Moreover, the volume of the cylinder is much smaller, thus consumes less amount of fluorescent fluid. What is more, the cylinder

design is more convenient for manufacturing and cleaning. Based on the above considerations, the cylindrical light channel was finally selected for subsequent research.

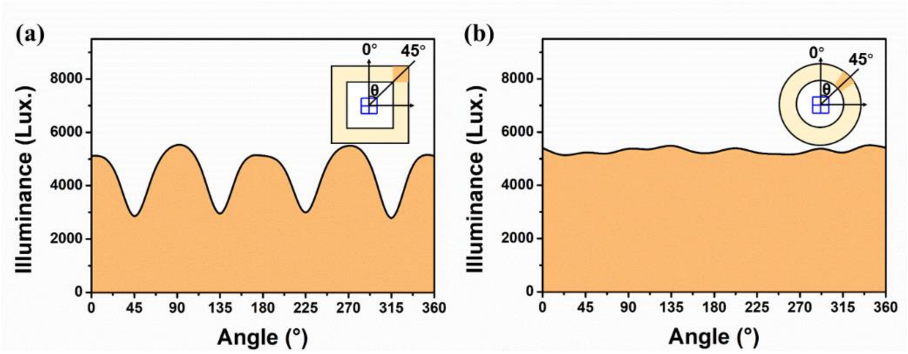

Figure S6. The distribution of illuminance varies with the angle where the unit light channel placed: (a) square channel and (b) cylindrical channel.

3. Experiment set-up

Experiments in Figure S7 was used to evaluate the performance of the photoreactor in general continuous-flow photochemical reaction. The connection between reactor channels and the reactant syringe is realized through perfluoroalkoxy (PFA) taper joint and PFA tubing with "1/16" outer diameter. Reaction solution A and B was injected to the reactor by two microinjection pumps. Moreover, the product was collected by a 4 mL centrifuge tube for UV-vis analysis. This tube was also wrapped in aluminum foil to prevent further conversion. The reactor was placed in the center of a reactor holder which was fabricated by the 3D print technique with polylactic acid as raw material. LED strips were wrapped around the inner wall of the cylinder holder so that the reactor can receive uniform light illumination. The photos of closed and open reactor holder were shown in Figure S8, the LED strips used in this experiment are 5050 model and 1meter long with standard voltage of 24 V. Their emission spectra were recorded in Figure S9. LED strips were powered with a high voltage power supply (GRALLEN, APS3003S-3D).

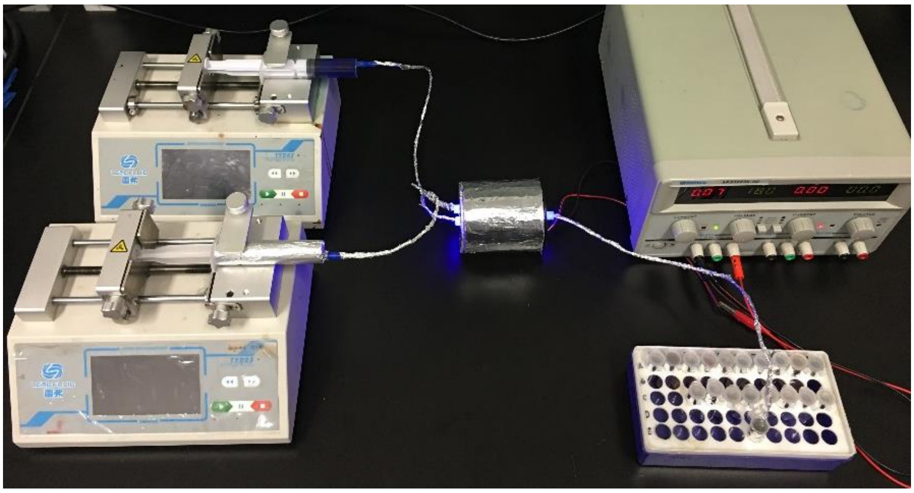

Figure S7. The experiment setup employed for reactor performance evaluation in a continuous-flow photochemical reaction.

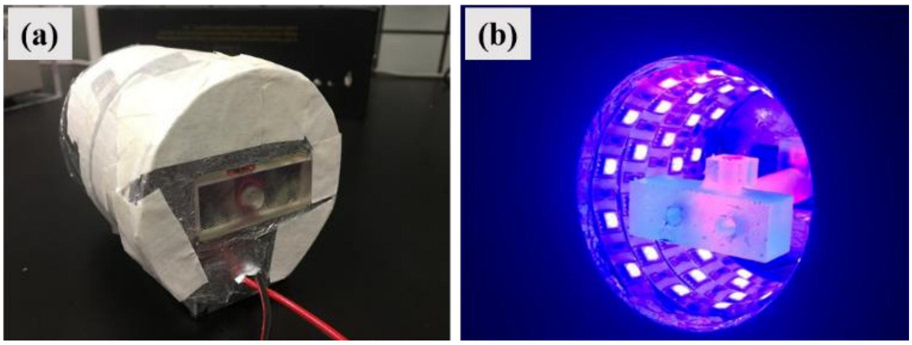

Figure S8. Blue LED reactor holder, closed (a) and open (b).

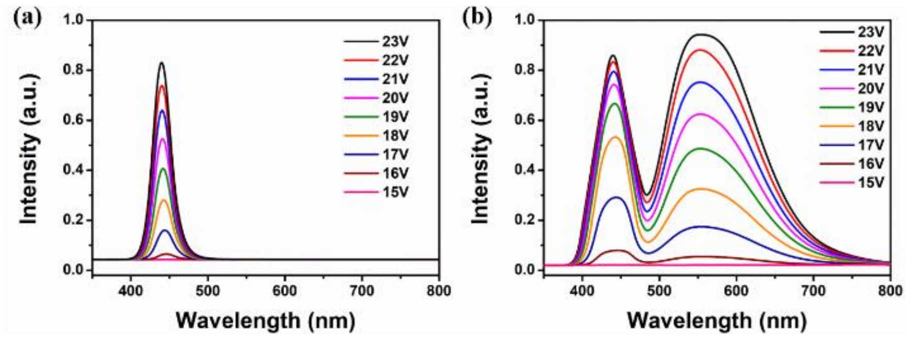

Figure S9. The emission spectrum of the blue LED strip (a) and white LED strip (b) with increased applied voltage. The nominal voltage is 24 V.

4. General reaction procedure

The residence time was calculated as below:

$$t = \frac{V_{reactionchannel}}{u_A + u_B} = \frac{R_{rout} \times R_{section}}{u_A + u_B}$$

where $V_{reaction}$ is the volume of reaction channel since the reaction solution A and B mix together, u_A and u_B are the volumetric flow rate of substrates, R_{rout} and $R_{section}$ are the routing length and section area of the reaction channel, respectively.

4.1 DPA oxidation reaction

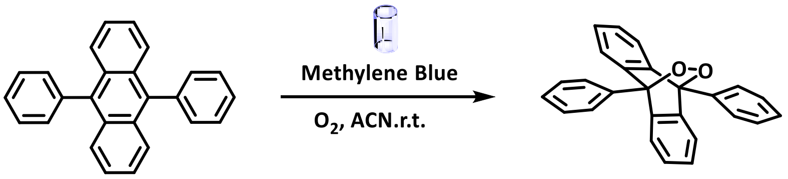

To conduct the oxidation of 1,9-diphenylanthracene (DPA), 100 mL stock solution of 1 mM DPA (>98%) and 2 mM methylene blue (MB, >98.5) in acetonitrile (ACN, >99.5%) are prepared. The DPA solution was stored under an argon atmosphere and wrapped in aluminum foil to protect from light. Before use, each storage solution 1.5 mL was taken out and diluted for 10 times, then loaded into a 20 mL syringe. Both DPA and MB were injected into the reaction channel through a syringe pump (Longer Pump Co. LTD) at a flow rate of 1:1. The syringe and PFA tubing with DPA solution outside the reactor were protected with aluminum foil to avoid conversion occurrence outside the reactor, while the MB solution was oxygen for 10 mins before it was injected. The product was collected with a 4 mL centrifuge tube, and the yield was measured with an UV-visible spectrometer (PERSEE, TU1900). The maximum absorption peak of DPA is located at 372 nm, and the conversion of DPA can be calculated according to the decrease in absorbance with the following equation:

$$\eta(\%) = \frac{Abs_{(DPA+MB)}(0) - Abs_{(DPA+MB)}(x)}{Abs_{(DPA+MB)}(0) - Abs_{MB}(0)} \times 100\%$$

Where the η (%) is the conversion rate, and $Abs_{DPA+MB}(0)$ and $Abs_{DPA+MB}(x)$ are the initial and residual absorbance of the mixed substrate solution, respectively. The $Abs_{MB}(0)$ is the initial absorbance of the MB solution. The absorption spectra of the substrate solution varies with the flow rate was showed in Figure S10. The product was confirmed by the Mass Spectrometry as shown in Figure S11.

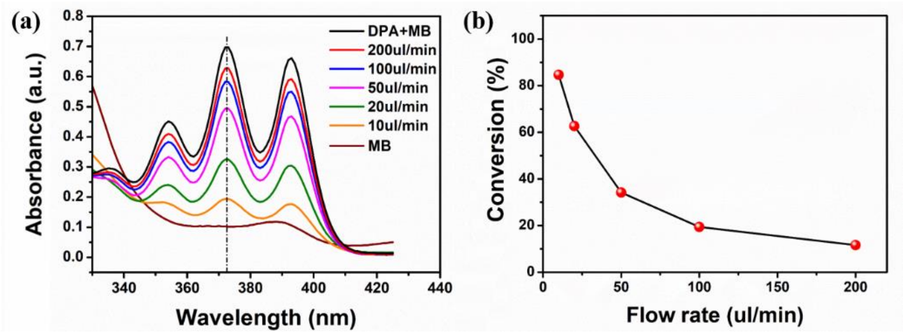

Figure S10. The absorption spectra (a) of reactants and their conversions (b) calculated according to the change in absorbance at 372nm at the different flow rates.

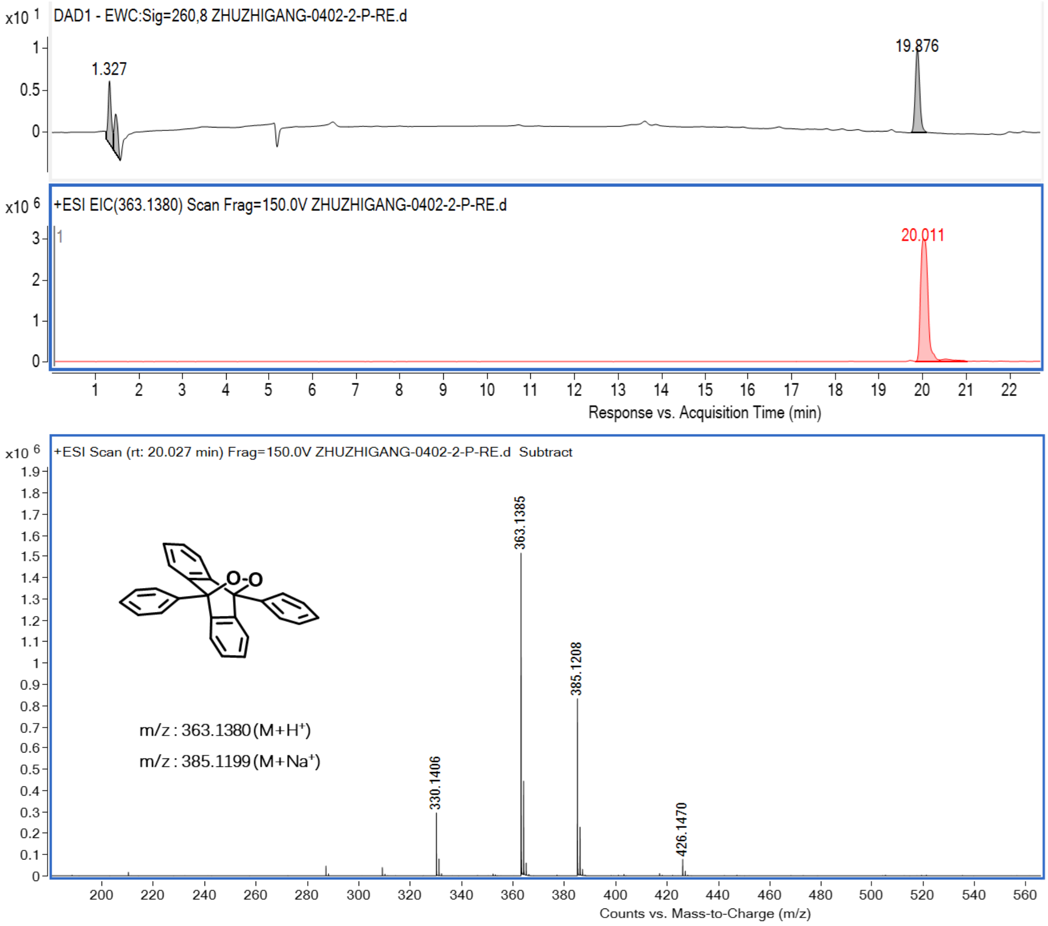

Figure S11. LC-TOF-MS of DPA oxidation products, which indicates that almost no other byproducts were produced.

Study on the effect of temperature on DPA conversion

Long-time working of the LED strip will cause a temperature increase over the reactor. To certify that the variation in reactor temperature does not affect the conversion of DPA, comparative experiments in a flask and our printed reactor were both carried out (Figure S12). The flask reaction was conducted under different temperature for 30 min, with DPA and MB mixed and stirred under dark condition. The conversion rate of DPA was very low about less than 1% and slightly decreased with temperature increasing from 30° to 90°, which shows that the temperature increase has no effect on reaction process promotion. For the reaction in our FFPM, DPA and MB were injected into the reaction channel at 50 uL/min, while the light channel kept empty. Then the reactor was placed in the center of LED strip holder, and the voltage of LED strip set as 22 V. During the reaction, 1ml product was taken every half hour for testing, and the surface temperature of the reactor was recorded with an infrared camera, results in Figure S12b shows that the surface temperature of the reactor increased from room temperature to 42 ℃ after 4 hours, and the conversion of DPA remained stable with only a slight rise.

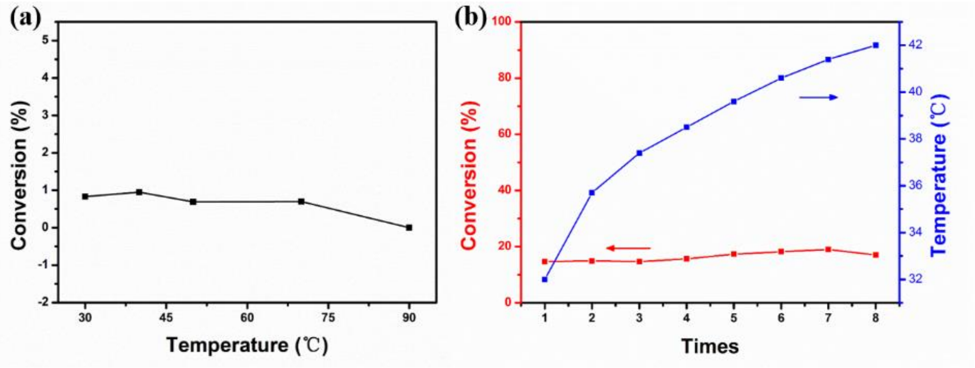

Figure S12. (a) The influence of temperature on the conversion of DPA in the flask. (b) Microreactor surface temperature fluctuation and conversion rate stability during the reaction;

Study on the effect of the back-reflection inside the reactor holder

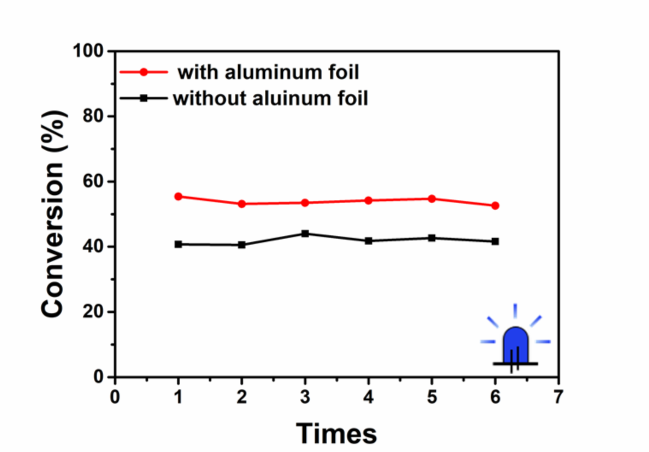

Figure S13. The comparison experiment of reactor holder with and without aluminum foil.

The comparison experiment of reactor holder with and without aluminum foil was conducted, the results confirmed that the presence of back-reflections does influence the reactor system, and the DPA conversion rate of the holder with aluminum foil is increased about 10%.

Experiment results in the serpentine reactor

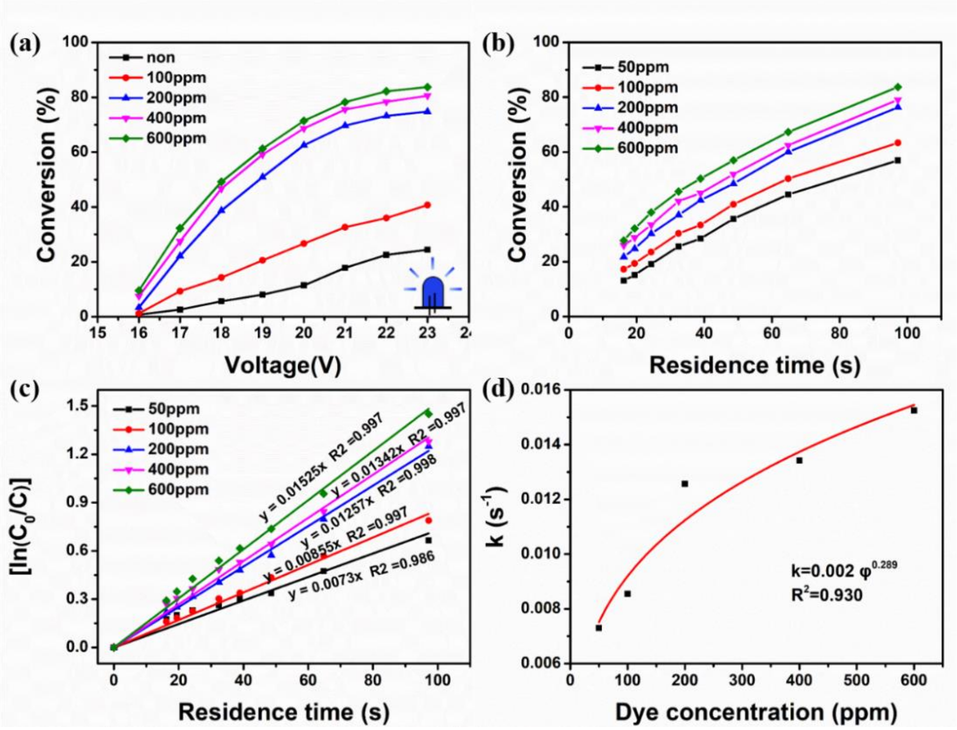

Figure S14. DPA conversion and reaction kinetic mechanism research in FFPM with the serpentine channel.

Experiment under white LED irradiation

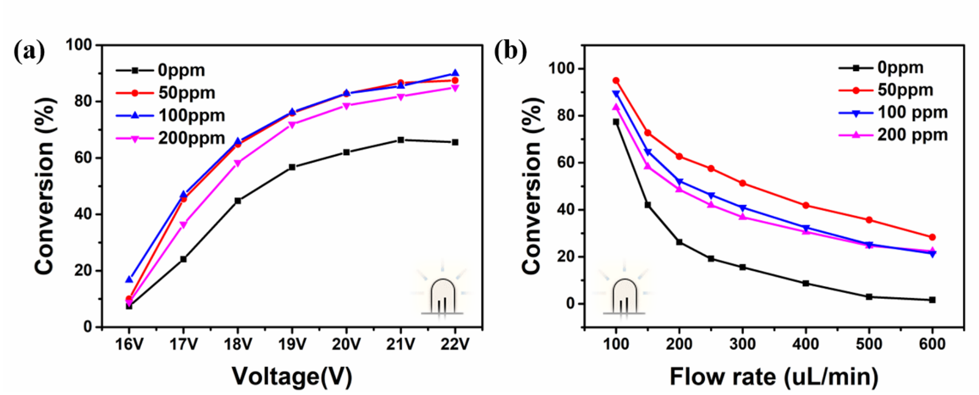

Figure S15. DPA conversion research in optimum FFPM under white LED irradiation (a) different voltage and (b) different flow rate.

Experiment under a simulated solar light source

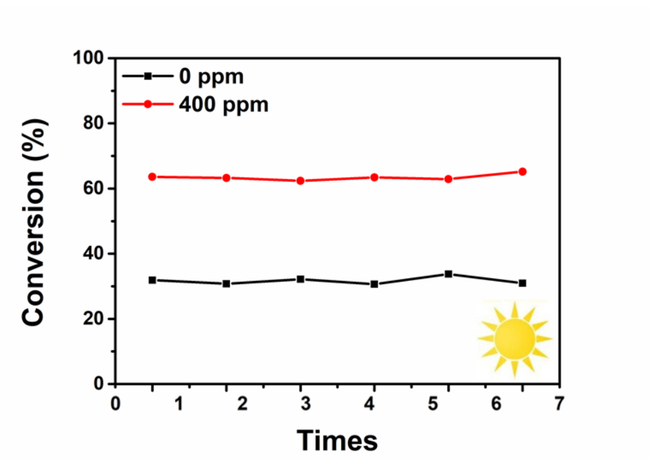

Figure S16. The performance of our FFPM under 1.0 sun illumination.(400 ppm LR305, residence time 15s)

We also conducted the experiment in which the FFPM is illuminated from one side in the air with a simulated solar light source (CEL-HXF300). This experiment was carried out on the desk with a black top, to eliminate the influence of desktop reflection as much as possible. The results show that the DPA conversion in FFPM with 400 ppm LR305 was significantly higher than FFPM with 0 ppm, under 1.0 sun illumination at a residence time of 15s. The presence of fluorescent fluids increases the conversion rate about 30%, which was comparable to the increase reported by Debije and Noel in Angew. Chem. Int. Ed. 2016, 55, 1.

Experiment by using different light-converting media

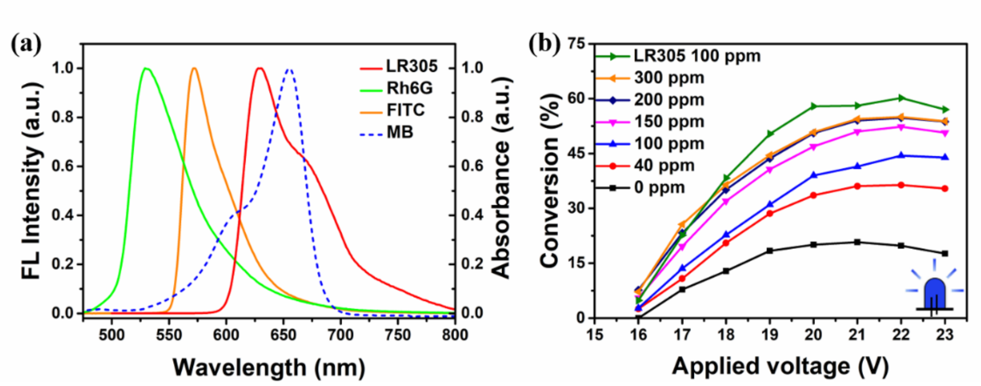

Figure S17. (a) Spectra overlapping scheme of different dyes emission to MB absorption, different dyes have almost equal fluorescence intensity, FITC: 37 ppm, Rh6G: 38 ppm, Eosin Y: 126 ppm, LR305: 100 ppm. (b) Conversion enhanced by different Rh6G concentration.

The transmission of emission light

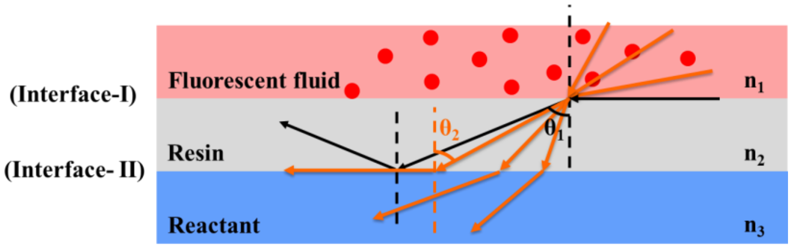

Figure S18. The transmittance behavior of emission light from fluorescent fluid to the reaction liquid.

To elucidate the transmittance behavior of emission light from fluorescent fluid to the reactants, we measured the effective refractive index of the fluorescent fluid and the reaction mixture, and combined with the refractive index of resin, gave a detailed explanation on the transmission process of emitted light.

In short, when the emission light is transmitted at the interface, it will be reflected and refracted, and only the refracted light can continue to propagate. Here, we discuss the refracted part. There are two interfaces from fluorescence fluid to reaction liquid, i.e. the interface between fluorescence fluid and resin (denote as I), and the interface between resin and reaction liquid (denote as II). The effective refractive index for the ethanol solution of LR305, resin and reaction liquid are 1.361, 1.512 and 1.343, respectively. Since $n_1 < n_2$, all

the light emitted from the fluorescence fluid can be refracted into the resin layer, while $n_2 > n_3$, the light may be fully reflected at the interface II, and only the light whose incident angle (θ_i) less than the critical angle (θ_2) can enter the reaction liquid. The critical angles of the two interfaces are calculated as follows:

$$\theta_1 = acrsin(\frac{n_1}{n_2}) \quad ; \quad \theta_2 = acrsin(\frac{n_3}{n_2})$$

After calculation, we can get θ_1= 64.17 °, θ_2= 62.65 °. Then only the light with 62.65 < θ_i < 64.17 cannot enter the reaction solution, the utilization rate of refracted light can reach 97.6%.

4.2 Thiophenol oxidation Reaction

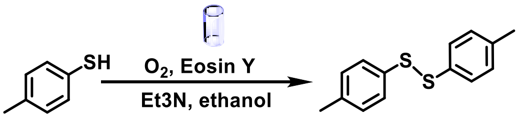

To conduct the oxidation reaction of p-thiocresol, 0.5 M p-thiocresol in ethanol with an internal standard (1,3,5-Trimethylbenzene, 0.01 mol) for GC analysis was prepared then transferred to a 20 mL syringe, which was wrapped with aluminum foil to prevent the conversion of p-thiocresol. At the same time, 0.5 M triethylamine in ethanol with 3 mol% Eosin Y as catalyst solution was transferred to another 20 mL syringe. These two solutions were introduced into our FFPM system by two syringe pumps at a flow rate of 50 uL/min. During the whole experiment, the working voltage of LED strip was set as 22 V, and the light channel was filled with FITC of different concentration (0, 100, 200, 400 ppm) which served as light harvest and wavelength conversion medium. The product was collected and purified by column chromatography on silica gel (eluent: ethyl acetate), then analyzed by GC-MS to obtain the p-thiocresol conversion.

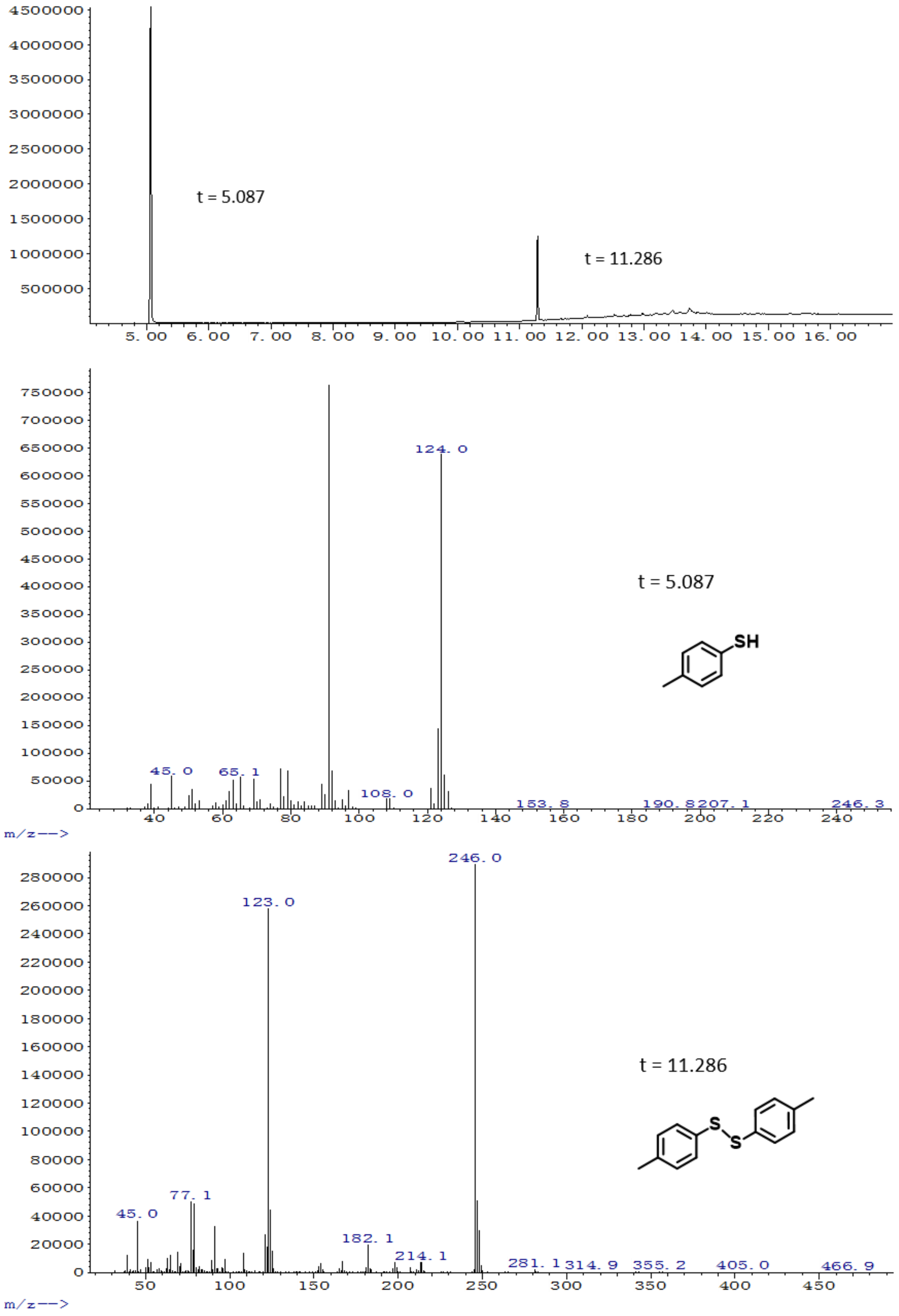

Figure S19. Liquid chromatography-tandem mass spectrometry for the product of p-thiocresol oxidation.

5. Table information

Table S1. The structural parameters of photomicroreactors with different optical channels.

		Helix	LA-2	LA-4	Cylinder
Microreactor Type		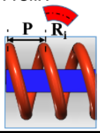	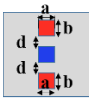	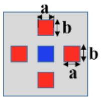	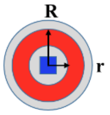
Light channel	Section	R_i = 2 mm<br>P = 8 mm	a = 1 mm<br>b = 1 mm	a = 1 mm<br>b = 1 mm	R = 2.5 mm<br>r = 1.5 mm
	Length	L = 105 mm	L = 56 mm	L = 56 mm	L = 56 mm
	volume	0.082 ml	0.112 ml	0.224 ml	0.704 ml
Resin layer thickness		d = 1 mm	d = 1 mm	d = 1 mm	d = 1 mm
Reactionchannel (Y type)	Section	1 mm x 1 mm	1 mm x 1 mm	1 mm x 1 mm	1 mm x 1 mm
	Length	56 mm	56 mm	56 mm	56 mm

Table S2. The structural parameters of photomicroreactors with different reaction channels.

		Y-Type	Helix-6	Helix-9	Helix-12
Microreactor Type		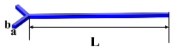	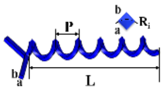	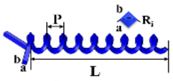	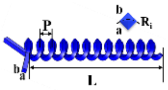
Reaction channel	Section	a = 1 mm<br>b = 1 mm	a = 1 mm<br>b = 1 mm	a = 1 mm<br>b = 1 mm	a = 1 mm<br>b = 1 mm
	Projection	1 mmx1 mm<br>——	R_i = 1.5 mm<br>P = 9.3 mm	R_i = 1.5 mm<br>P = 6.2 mm	R_i = 1.5 mm<br>P = 4.7 mm
	Rout	S = 56 mm	S = 80 mm	S = 102 mm	S = 126 mm
Resin layer thickness		d = 1 mm	d = 1 mm	d = 1 mm	d = 1 mm
Light channel (Cylinder)	Section	R = 4 mm,<br>r = 3 mm	R = 4 mm,<br>r = 3 mm	R = 4 mm,<br>r = 3 mm	R = 4 mm,<br>r = 3 mm
	Length	L = 56 mm	L = 56 mm	L = 56 mm	L = 56 mm
	Volume	1.23 ml	1.23 ml	1.23 ml	1.23 ml

Table S3. The price parameters of the LED lamp with different wavelength (Power: 3W).

Color	Wavelength (nm)	Efficiency (lm/W)	Luminous Angle (°)	Working Current (mA)	Working Voltage (V)	Price/100 ($)
Blue	440-460	90	120	700	3	14.1
Red	660	90	120	700	3	19.2
Green	520-525	90	120	700	3	25.2
Yellow	588-590	90	120	700	3	26.6
Orange	597-600	90	120	700	3	31.1
Pink	----	90	120	700	3	34.0
Purple	395-400	90	120	700	3	47.4
White	RGB	90	120	700	3	47.4

Table S4. The price parameters for different light-converting media.[a)]

Name	CAS	Quality	Purity	Price($)
Lumogen F Red 305	123174-58-3	1 g	>95.0%(N)	92.5
Coumarin 6	38215-36-0	1 g	>98.0%(HPLC)	76.4
Coumarin 153	53518-18-6	1 g	>98.0%(HPLC)	374.2
Coumarin 314	55804-66-5	1 g	>98.0%(HPLC)	199.8
Rubrene	517-51-1	1 g	>98.0%(HPLC)	89.3
Zinc(II) (TPBP)	14074-80-7	1 g	>98.0%(HPLC)	119.1
DCM	51325-91-8	1 g	95%	297.8
DCJTB	200052-70-6	0.2 g	>98.0%(HPLC)	226.6
FITC	27072-45-3	1g	95%, A mixture of 5- and 6- [illegible]	147.4
Rhodamine B	81-88-9	100 g	AR	14.8
Rhodamine 6G	989-38-8	100 g	95%	89.3
Eosin Y	17372-87-1	100g	AR	132.6
PbS QD	----	50 mg	----	590.1
ZnSe/ZnS	----	5 ml	1 mg/mL in Toluene	215.9
CdSe/ZnS QD	1306-24-7	5 mL	12 μmol/L in Cyclohexane	469.2
CdSe/ZnS QD	1306-24-7	1 mL	8 μmol/L in H2O	938.6
Perovskite QD	----	1 ml	40-50 mg/mL	178.7

[a)]The quotation is from the official website of Aladdin reagent in China.

Table S5. The tolerance of transparent resin to different solvents. (A resin slice of 20 mmx40 mmx1mm was thoroughly immersed in the solvent.)

Solvent \ Time	12.5h	24.5h	95.5h	Photos
Water	√	√	√	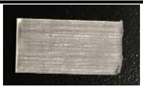
Toluene	√	√	√	
Isopropanol	√	√	√	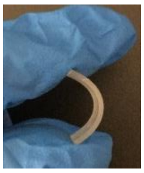
Ethyl acetate	√	√	√	
Ethanol	√	√	√	
Acetonitrile	√	√	√	
Methanol	√	√	×	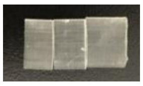
acetic acid	√	√	×	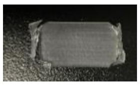
Dichloromethane	√	×	----	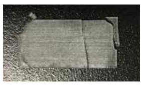
DMF	√	×	----	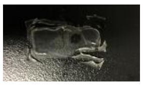
